# 2,2′-[(Propane-1,3-diyldinitrilo)bis­(phenyl­methyl­idyne)]diphenol

**DOI:** 10.1107/S1600536810011189

**Published:** 2010-03-31

**Authors:** Robert S. Black, David G. Billing, Agata Bartyzel, Ewa Cukrowska

**Affiliations:** a Molecular Sciences Institute, School of Chemistry, University of the Witwatersrand, Private Bag 3, PO Wits 2050, South Africa

## Abstract

In the title mol­ecule, C_29_H_26_N_2_O_2_, there are two strong intra­molecular O—H⋯N hydrogen bonds involving the hydr­oxy and imine groups, forming *S*(6) ring motifs. The dihedral angles between adjacent phenyl rings and phenol-containing planes are 85.27 (19) and 91.38 (18)°. In the crystal structure, weak inter­molecular C—H⋯O hydrogen bonds connect mol­ecules into a two-dimensional network.

## Related literature

The title compound forms part of the group of Schiff bases with a similar method of synthesis as described in Schilf *et al.* (2007 ▶). The inter­molecular hydrogen bonds O—H⋯N between the hydr­oxy and imine are common to this type of compound as shown with the series of compounds reported by Fernández *et al.* (2001 ▶); Kabak (2003 ▶); Wojciechowski *et al.* (2001 ▶); Dey *et al.* (2001 ▶); Koşar, *et al.* (2004 ▶); Lu, *et al.* (2008 ▶); Qiu & Zhao (2008 ▶); Montazerozohori *et al.* (2009 ▶); Corden *et al.* (1996 ▶). For a decription of hydrogen-bond motifs, see: Bernstein *et al.* (1995 ▶).
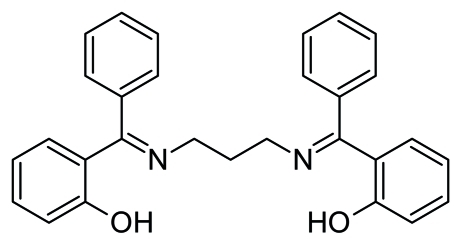


## Experimental

### 

#### Crystal data

C_29_H_26_N_2_O_2_
*M* *_r_* = 434.52
Monoclinic, 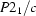

*a* = 18.226 (2) Å
*b* = 8.2303 (9) Å
*c* = 18.642 (2) Å
β = 119.086 (5)°
*V* = 2443.7 (5) Å^3^
*Z* = 4
Mo *K*α radiation
μ = 0.07 mm^−1^
*T* = 293 K
0.7 × 0.5 × 0.28 mm

#### Data collection

Bruker SMART 1K CCD area-detector diffractometer
21350 measured reflections
5896 independent reflections
3749 reflections with *I* > 2σ(*I*)
*R*_int_ = 0.071

#### Refinement

*R*[*F* ^2^ > 2σ(*F* ^2^)] = 0.055
*wR*(*F* ^2^) = 0.173
*S* = 1.03
5896 reflections
300 parameters
H-atom parameters constrained
Δρ_max_ = 0.23 e Å^−3^
Δρ_min_ = −0.36 e Å^−3^

### 

Data collection: *SMART-NT* (Bruker, 1998 ▶); cell refinement: *SAINT-Plus* (Bruker, 1999 ▶); data reduction: *SAINT-Plus*; program(s) used to solve structure: *SHELXS97* (Sheldrick, 2008 ▶); program(s) used to refine structure: *SHELXL97* (Sheldrick, 2008 ▶); molecular graphics: *PLATON* (Spek, 2009 ▶); software used to prepare material for publication: *WinGX* (Farrugia, 1999 ▶) and *PLATON*.

## Supplementary Material

Crystal structure: contains datablocks global, I. DOI: 10.1107/S1600536810011189/lh5012sup1.cif

Structure factors: contains datablocks I. DOI: 10.1107/S1600536810011189/lh5012Isup2.hkl

Additional supplementary materials:  crystallographic information; 3D view; checkCIF report

## Figures and Tables

**Table 1:** Hydrogen-bond geometry (Å, °)

*D*—H⋯*A*	*D*—H	H⋯*A*	*D*⋯*A*	*D*—H⋯*A*
O1—H1*A*⋯N1	0.82	1.80	2.5344 (17)	148
O2—H2*A*⋯N2	0.82	1.81	2.5431 (18)	148
C17—H17⋯O2^i^	0.93	2.56	3.481 (2)	168
C18—H18⋯O1^ii^	0.93	2.47	3.395 (3)	174
C21—H21⋯O2^iii^	0.93	2.56	3.492 (2)	175

Symmetry codes: (i) 
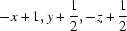
; (ii) 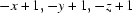; (iii) 
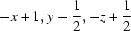
.

**Fig. 1.**
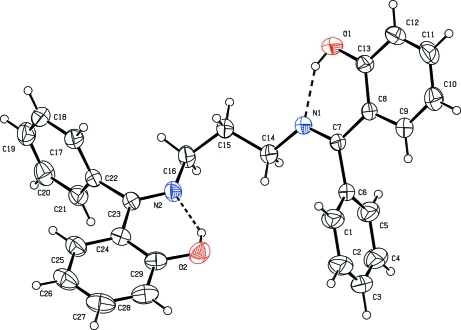
The molecular structure of the title compound with displacement ellipsoids drawn at the 30% probability level. Hydrogen bonds are shown as dashed lines.

## supplementary crystallographic information

### Comment

The molecular structure of the title compound is shown in Fig. 1. There are two strong intramolecular O—H..O hydrogen bonds involving the hydroxyl and imine groups forming S(6) ring motifs (Bernstein *et al.*, 1995). These types of hydrogen bonds are common to some reported molecular structures (Schilf *et al.*, 2007; Fernández *et al.*, 2001; Kabak, 2003; Wojciechowski *et al.*, 2001; Dey *et al.*, 2001; Koşar, *et al.*, 2004; Lu, *et al.*, 2008; Qiu & Zhao, 2008; Montazerozohori *et al., *2009; Corden *et al.*, 1996). In the crystal structure, weak intermolecular C—H···O hydrogen bond connect molecules to form a two-dimensional network.

### Experimental

A mixture of 1.0 mmol (2.00 g) of 2-hydroxybenzophenone, 0.5 mmole (0.42 cm^3^) of 1,3-propanediamine and 2 drops of glacial acetic acid in 40 ml of methanol was refluxed for 8 h. The excess of solvent (ca. 30 ml) was then evaporated. After cooling to 277 K a yellow solid was produced. The polycrystalline product was collected by filtration, washed with methanol and dried. A yield of 52% was obtained. Recrystalization from an ethanol solution yielded single crystals suitable for x-ray diffraction. Elemental analysis *C% 79.67 H% 6.26 N% 6.11%*.

### Refinement

All H atoms were refined using a riding model, with C—H = 0.93-0.97 Å, O—H = 0.82 Å, and *U*iso(H) = 1.2*U*eq(C) or 1.5*U*eq(O).

### Crystal data

**Table:**

C_29_H_26_N_2_O_2_	*F*(000) = 920
*M**_r_* = 434.52	*D*_x_ = 1.181 Mg m^−^^3^
Monoclinic, *P*2_1_/*c*	Mo *K*α radiation, λ = 0.71073 Å
Hall symbol: -P 2ybc	Cell parameters from 21350 reflections
*a* = 18.226 (2) Å	θ = 2.1–23.0°
*b* = 8.2303 (9) Å	µ = 0.07 mm^−^^1^
*c* = 18.642 (2) Å	*T* = 293 K
β = 119.086 (5)°	Block, yellow
*V* = 2443.7 (5) Å^3^	0.7 × 0.5 × 0.28 mm
*Z* = 4	

### Data collection

**Table:**

Bruker SMART 1K CCD area-detector diffractometer	*R*_int_ = 0.071
φ and ω scans	θ_max_ = 28°, θ_min_ = 1.3°
21350 measured reflections	*h* = −19→24
5896 independent reflections	*k* = −10→10
3749 reflections with *I* > 2σ(*I*)	*l* = −24→20

### Refinement

**Table:**

Refinement on *F*^2^	0 restraints
Least-squares matrix: full	H-atom parameters constrained
*R*[*F*^2^ > 2σ(*F*^2^)] = 0.055	*w* = 1/[σ^2^(*F*_o_^2^) + (0.0897*P*)^2^ + 0.2258*P*] where *P* = (*F*_o_^2^ + 2*F*_c_^2^)/3
*wR*(*F*^2^) = 0.173	(Δ/σ)_max_ = 0.002
*S* = 1.03	Δρ_max_ = 0.23 e Å^−^^3^
5896 reflections	Δρ_min_ = −0.36 e Å^−^^3^
300 parameters	

### Special details

**Table:**

Geometry. All esds (except the esd in the dihedral angle between two l.s. planes) are estimated using the full covariance matrix. The cell esds are taken into account individually in the estimation of esds in distances, angles and torsion angles; correlations between esds in cell parameters are only used when they are defined by crystal symmetry. An approximate (isotropic) treatment of cell esds is used for estimating esds involving l.s. planes.

### Fractional atomic coordinates and isotropic or equivalent isotropic displacement parameters (Å^2^)

**Table:**

	*x*	*y*	*z*	*U*_iso_*/*U*_eq_	
C1	0.17087 (13)	0.1655 (3)	0.08228 (11)	0.0718 (5)	
H1	0.1926	0.2672	0.0813	0.086*	
C2	0.15625 (16)	0.0517 (3)	0.02117 (14)	0.0940 (7)	
H2	0.1689	0.0776	−0.0201	0.113*	
C3	0.12367 (14)	−0.0961 (3)	0.02162 (16)	0.0947 (8)	
H3	0.1142	−0.1708	−0.0194	0.114*	
C4	0.10480 (16)	−0.1362 (3)	0.08146 (16)	0.0960 (7)	
H4	0.0823	−0.2377	0.0812	0.115*	
C5	0.11925 (13)	−0.0247 (3)	0.14328 (13)	0.0759 (5)	
H5	0.1062	−0.0519	0.1842	0.091*	
C6	0.15300 (9)	0.12641 (19)	0.14400 (9)	0.0501 (4)	
C7	0.17057 (8)	0.24201 (18)	0.21320 (9)	0.0457 (3)	
C8	0.10782 (9)	0.36732 (18)	0.20194 (9)	0.0476 (3)	
C9	0.03699 (10)	0.3944 (2)	0.12497 (11)	0.0612 (4)	
H9	0.0291	0.3308	0.0805	0.073*	
C10	−0.02150 (12)	0.5138 (3)	0.11365 (13)	0.0775 (6)	
H10	−0.0675	0.5312	0.062	0.093*	
C11	−0.01072 (13)	0.6066 (3)	0.17994 (16)	0.0826 (6)	
H11	−0.05	0.6861	0.1726	0.099*	
C12	0.05729 (13)	0.5829 (2)	0.25660 (14)	0.0735 (5)	
H12	0.0632	0.6461	0.3004	0.088*	
C13	0.11810 (10)	0.4642 (2)	0.26941 (10)	0.0548 (4)	
C14	0.30624 (10)	0.1168 (2)	0.29798 (11)	0.0637 (4)	
H14A	0.3103	0.1037	0.2483	0.076*	
H14B	0.2935	0.0117	0.3128	0.076*	
C15	0.38907 (10)	0.1788 (2)	0.36662 (10)	0.0590 (4)	
H15A	0.385	0.1869	0.4165	0.071*	
H15B	0.3992	0.287	0.3528	0.071*	
C16	0.46364 (10)	0.0702 (2)	0.38294 (10)	0.0606 (4)	
H16A	0.5125	0.1034	0.4336	0.073*	
H16B	0.4508	−0.0416	0.3891	0.073*	
C17	0.66693 (10)	0.2305 (2)	0.45511 (10)	0.0602 (4)	
H17	0.6442	0.3306	0.4316	0.072*	
C18	0.73422 (12)	0.2233 (3)	0.53470 (12)	0.0729 (5)	
H18	0.756	0.3185	0.5644	0.087*	
C19	0.76854 (12)	0.0763 (3)	0.56945 (12)	0.0743 (5)	
H19	0.813	0.0721	0.6229	0.089*	
C20	0.73734 (12)	−0.0646 (3)	0.52550 (13)	0.0747 (5)	
H20	0.7618	−0.1637	0.5489	0.09*	
C21	0.66949 (11)	−0.0604 (2)	0.44627 (11)	0.0608 (4)	
H21	0.6481	−0.1564	0.4172	0.073*	
C22	0.63373 (9)	0.08832 (18)	0.41069 (9)	0.0469 (3)	
C23	0.55780 (9)	0.09263 (18)	0.32620 (9)	0.0496 (4)	
C24	0.57020 (11)	0.10567 (19)	0.25341 (10)	0.0539 (4)	
C25	0.65069 (12)	0.1279 (2)	0.26229 (12)	0.0676 (5)	
H25	0.6968	0.134	0.3147	0.081*	
C26	0.66269 (15)	0.1409 (3)	0.19441 (15)	0.0808 (6)	
H26	0.7163	0.156	0.2012	0.097*	
C27	0.59361 (18)	0.1312 (3)	0.11627 (15)	0.0861 (7)	
H27	0.6013	0.1387	0.0706	0.103*	
C28	0.51399 (16)	0.1106 (2)	0.10547 (12)	0.0769 (6)	
H28	0.4686	0.1045	0.0527	0.092*	
C29	0.50056 (12)	0.09859 (19)	0.17301 (11)	0.0597 (4)	
N1	0.23864 (8)	0.23289 (17)	0.28316 (8)	0.0562 (3)	
N2	0.48206 (8)	0.08218 (17)	0.31467 (8)	0.0564 (3)	
O1	0.18408 (8)	0.44387 (17)	0.34460 (7)	0.0709 (4)	
H1A	0.2167	0.3781	0.3424	0.106*	
O2	0.42130 (8)	0.08050 (17)	0.15964 (7)	0.0715 (4)	
H2A	0.4218	0.074	0.2038	0.107*	

### Atomic displacement parameters (Å^2^)

**Table:**

	*U*^11^	*U*^22^	*U*^33^	*U*^12^	*U*^13^	*U*^23^
C1	0.0820 (13)	0.0800 (12)	0.0680 (11)	−0.0146 (10)	0.0478 (10)	−0.0153 (9)
C2	0.1009 (17)	0.125 (2)	0.0795 (14)	−0.0200 (15)	0.0625 (14)	−0.0331 (13)
C3	0.0776 (14)	0.1138 (19)	0.1001 (16)	−0.0262 (13)	0.0490 (13)	−0.0609 (14)
C4	0.0966 (17)	0.0856 (15)	0.1172 (18)	−0.0384 (13)	0.0610 (16)	−0.0465 (14)
C5	0.0807 (13)	0.0769 (12)	0.0816 (13)	−0.0279 (10)	0.0484 (11)	−0.0252 (10)
C6	0.0390 (7)	0.0596 (9)	0.0508 (8)	−0.0030 (6)	0.0212 (6)	−0.0107 (7)
C7	0.0393 (7)	0.0540 (8)	0.0465 (7)	−0.0056 (6)	0.0229 (6)	−0.0073 (6)
C8	0.0400 (7)	0.0531 (8)	0.0529 (8)	−0.0062 (6)	0.0250 (7)	−0.0070 (6)
C9	0.0456 (9)	0.0714 (11)	0.0615 (10)	−0.0002 (7)	0.0220 (8)	−0.0086 (8)
C10	0.0513 (10)	0.0855 (13)	0.0823 (13)	0.0124 (9)	0.0221 (9)	−0.0007 (11)
C11	0.0607 (12)	0.0732 (12)	0.1165 (18)	0.0144 (9)	0.0451 (13)	−0.0065 (12)
C12	0.0679 (12)	0.0709 (12)	0.0926 (14)	0.0000 (9)	0.0475 (11)	−0.0229 (10)
C13	0.0520 (9)	0.0573 (9)	0.0625 (10)	−0.0083 (7)	0.0338 (8)	−0.0127 (7)
C14	0.0450 (9)	0.0724 (11)	0.0627 (10)	0.0065 (7)	0.0175 (8)	−0.0136 (8)
C15	0.0442 (8)	0.0750 (11)	0.0546 (9)	−0.0015 (7)	0.0217 (7)	−0.0083 (8)
C16	0.0435 (8)	0.0848 (12)	0.0528 (9)	0.0052 (8)	0.0229 (7)	0.0092 (8)
C17	0.0553 (9)	0.0568 (9)	0.0662 (10)	−0.0005 (7)	0.0277 (8)	−0.0048 (8)
C18	0.0569 (10)	0.0913 (14)	0.0704 (12)	−0.0188 (10)	0.0310 (9)	−0.0281 (10)
C19	0.0501 (10)	0.1127 (17)	0.0548 (10)	0.0029 (10)	0.0213 (8)	0.0014 (10)
C20	0.0621 (11)	0.0852 (14)	0.0726 (12)	0.0204 (10)	0.0294 (10)	0.0230 (11)
C21	0.0566 (10)	0.0567 (9)	0.0669 (10)	0.0052 (7)	0.0282 (9)	0.0009 (8)
C22	0.0402 (7)	0.0523 (8)	0.0505 (8)	0.0024 (6)	0.0237 (7)	−0.0007 (6)
C23	0.0455 (8)	0.0508 (8)	0.0518 (8)	0.0035 (6)	0.0230 (7)	−0.0005 (6)
C24	0.0605 (9)	0.0511 (8)	0.0554 (9)	0.0042 (7)	0.0324 (8)	−0.0011 (7)
C25	0.0690 (11)	0.0714 (11)	0.0764 (12)	0.0021 (9)	0.0465 (10)	−0.0025 (9)
C26	0.0994 (16)	0.0749 (13)	0.1028 (16)	0.0019 (11)	0.0763 (15)	−0.0001 (11)
C27	0.136 (2)	0.0732 (13)	0.0831 (15)	0.0020 (13)	0.0797 (16)	0.0013 (11)
C28	0.1119 (17)	0.0633 (11)	0.0601 (11)	0.0001 (11)	0.0456 (12)	−0.0017 (8)
C29	0.0754 (12)	0.0501 (9)	0.0562 (9)	0.0028 (8)	0.0340 (9)	−0.0002 (7)
N1	0.0429 (7)	0.0685 (9)	0.0517 (7)	0.0042 (6)	0.0187 (6)	−0.0111 (6)
N2	0.0442 (7)	0.0732 (9)	0.0494 (7)	0.0036 (6)	0.0209 (6)	0.0030 (6)
O1	0.0677 (8)	0.0854 (9)	0.0572 (7)	0.0013 (6)	0.0285 (6)	−0.0219 (6)
O2	0.0667 (8)	0.0858 (9)	0.0505 (6)	−0.0029 (6)	0.0193 (6)	−0.0008 (6)

### Geometric parameters (Å, °)

**Table:**

C1—C6	1.378 (2)	C15—H15B	0.97
C1—C2	1.396 (3)	C16—N2	1.469 (2)
C1—H1	0.93	C16—H16A	0.97
C2—C3	1.356 (3)	C16—H16B	0.97
C2—H2	0.93	C17—C22	1.391 (2)
C3—C4	1.358 (3)	C17—C18	1.393 (3)
C3—H3	0.93	C17—H17	0.93
C4—C5	1.394 (3)	C18—C19	1.371 (3)
C4—H4	0.93	C18—H18	0.93
C5—C6	1.385 (2)	C19—C20	1.374 (3)
C5—H5	0.93	C19—H19	0.93
C6—C7	1.507 (2)	C20—C21	1.392 (3)
C7—N1	1.2934 (19)	C20—H20	0.93
C7—C8	1.477 (2)	C21—C22	1.394 (2)
C8—C9	1.405 (2)	C21—H21	0.93
C8—C13	1.422 (2)	C22—C23	1.509 (2)
C9—C10	1.389 (3)	C23—N2	1.293 (2)
C9—H9	0.93	C23—C24	1.484 (2)
C10—C11	1.383 (3)	C24—C25	1.406 (2)
C10—H10	0.93	C24—C29	1.418 (2)
C11—C12	1.376 (3)	C25—C26	1.389 (3)
C11—H11	0.93	C25—H25	0.93
C12—C13	1.408 (2)	C26—C27	1.389 (3)
C12—H12	0.93	C26—H26	0.93
C13—O1	1.342 (2)	C27—C28	1.375 (3)
C14—N1	1.475 (2)	C27—H27	0.93
C14—C15	1.516 (2)	C28—C29	1.398 (3)
C14—H14A	0.97	C28—H28	0.93
C14—H14B	0.97	C29—O2	1.349 (2)
C15—C16	1.528 (2)	O1—H1A	0.82
C15—H15A	0.97	O2—H2A	0.82
			
C6—C1—C2	119.74 (19)	H15A—C15—H15B	107.7
C6—C1—H1	120.1	N2—C16—C15	110.13 (13)
C2—C1—H1	120.1	N2—C16—H16A	109.6
C3—C2—C1	120.5 (2)	C15—C16—H16A	109.6
C3—C2—H2	119.8	N2—C16—H16B	109.6
C1—C2—H2	119.8	C15—C16—H16B	109.6
C2—C3—C4	120.63 (19)	H16A—C16—H16B	108.1
C2—C3—H3	119.7	C22—C17—C18	120.06 (16)
C4—C3—H3	119.7	C22—C17—H17	120
C3—C4—C5	119.9 (2)	C18—C17—H17	120
C3—C4—H4	120.1	C19—C18—C17	120.31 (18)
C5—C4—H4	120.1	C19—C18—H18	119.8
C6—C5—C4	120.23 (19)	C17—C18—H18	119.8
C6—C5—H5	119.9	C18—C19—C20	120.11 (17)
C4—C5—H5	119.9	C18—C19—H19	119.9
C1—C6—C5	119.07 (16)	C20—C19—H19	119.9
C1—C6—C7	121.98 (15)	C19—C20—C21	120.55 (17)
C5—C6—C7	118.94 (15)	C19—C20—H20	119.7
N1—C7—C8	118.72 (13)	C21—C20—H20	119.7
N1—C7—C6	121.82 (13)	C20—C21—C22	119.74 (16)
C8—C7—C6	119.45 (12)	C20—C21—H21	120.1
C9—C8—C13	118.36 (14)	C22—C21—H21	120.1
C9—C8—C7	121.28 (13)	C17—C22—C21	119.22 (15)
C13—C8—C7	120.37 (13)	C17—C22—C23	120.95 (13)
C10—C9—C8	121.58 (17)	C21—C22—C23	119.78 (13)
C10—C9—H9	119.2	N2—C23—C24	118.54 (14)
C8—C9—H9	119.2	N2—C23—C22	122.36 (14)
C11—C10—C9	119.33 (19)	C24—C23—C22	119.09 (13)
C11—C10—H10	120.3	C25—C24—C29	118.44 (16)
C9—C10—H10	120.3	C25—C24—C23	121.04 (15)
C12—C11—C10	120.99 (17)	C29—C24—C23	120.51 (15)
C12—C11—H11	119.5	C26—C25—C24	121.34 (19)
C10—C11—H11	119.5	C26—C25—H25	119.3
C11—C12—C13	120.75 (17)	C24—C25—H25	119.3
C11—C12—H12	119.6	C27—C26—C25	119.15 (19)
C13—C12—H12	119.6	C27—C26—H26	120.4
O1—C13—C12	119.70 (15)	C25—C26—H26	120.4
O1—C13—C8	121.33 (14)	C28—C27—C26	120.96 (18)
C12—C13—C8	118.98 (16)	C28—C27—H27	119.5
N1—C14—C15	109.80 (14)	C26—C27—H27	119.5
N1—C14—H14A	109.7	C27—C28—C29	120.7 (2)
C15—C14—H14A	109.7	C27—C28—H28	119.6
N1—C14—H14B	109.7	C29—C28—H28	119.6
C15—C14—H14B	109.7	O2—C29—C28	118.80 (17)
H14A—C14—H14B	108.2	O2—C29—C24	121.83 (15)
C14—C15—C16	113.32 (14)	C28—C29—C24	119.37 (18)
C14—C15—H15A	108.9	C7—N1—C14	122.06 (13)
C16—C15—H15A	108.9	C23—N2—C16	122.40 (13)
C14—C15—H15B	108.9	C13—O1—H1A	109.5
C16—C15—H15B	108.9	C29—O2—H2A	109.5
			
C6—C1—C2—C3	0.8 (4)	C18—C19—C20—C21	−1.6 (3)
C1—C2—C3—C4	−0.1 (4)	C19—C20—C21—C22	1.0 (3)
C2—C3—C4—C5	−0.2 (4)	C18—C17—C22—C21	−1.1 (2)
C3—C4—C5—C6	−0.2 (4)	C18—C17—C22—C23	176.55 (14)
C2—C1—C6—C5	−1.2 (3)	C20—C21—C22—C17	0.3 (2)
C2—C1—C6—C7	177.65 (19)	C20—C21—C22—C23	−177.36 (15)
C4—C5—C6—C1	0.9 (3)	C17—C22—C23—N2	−90.01 (19)
C4—C5—C6—C7	−177.97 (19)	C21—C22—C23—N2	87.61 (19)
C1—C6—C7—N1	−96.1 (2)	C17—C22—C23—C24	91.38 (18)
C5—C6—C7—N1	82.8 (2)	C21—C22—C23—C24	−91.00 (18)
C1—C6—C7—C8	85.27 (19)	N2—C23—C24—C25	175.86 (15)
C5—C6—C7—C8	−95.88 (19)	C22—C23—C24—C25	−5.5 (2)
N1—C7—C8—C9	173.25 (15)	N2—C23—C24—C29	−3.1 (2)
C6—C7—C8—C9	−8.1 (2)	C22—C23—C24—C29	175.57 (13)
N1—C7—C8—C13	−6.6 (2)	C29—C24—C25—C26	−0.7 (3)
C6—C7—C8—C13	172.08 (13)	C23—C24—C25—C26	−179.70 (16)
C13—C8—C9—C10	1.0 (3)	C24—C25—C26—C27	−0.2 (3)
C7—C8—C9—C10	−178.90 (17)	C25—C26—C27—C28	0.7 (3)
C8—C9—C10—C11	−1.2 (3)	C26—C27—C28—C29	−0.1 (3)
C9—C10—C11—C12	0.5 (3)	C27—C28—C29—O2	179.16 (17)
C10—C11—C12—C13	0.4 (3)	C27—C28—C29—C24	−0.9 (3)
C11—C12—C13—O1	179.67 (18)	C25—C24—C29—O2	−178.77 (16)
C11—C12—C13—C8	−0.6 (3)	C23—C24—C29—O2	0.2 (2)
C9—C8—C13—O1	179.63 (15)	C25—C24—C29—C28	1.2 (2)
C7—C8—C13—O1	−0.5 (2)	C23—C24—C29—C28	−179.77 (15)
C9—C8—C13—C12	−0.1 (2)	C8—C7—N1—C14	−176.84 (14)
C7—C8—C13—C12	179.80 (15)	C6—C7—N1—C14	4.5 (2)
N1—C14—C15—C16	−176.82 (14)	C15—C14—N1—C7	155.47 (15)
C14—C15—C16—N2	70.2 (2)	C24—C23—N2—C16	−179.47 (14)
C22—C17—C18—C19	0.5 (3)	C22—C23—N2—C16	1.9 (2)
C17—C18—C19—C20	0.8 (3)	C15—C16—N2—C23	137.49 (16)

### Hydrogen-bond geometry (Å, °)

**Table:**

*D*—H···*A*	*D*—H	H···*A*	*D*···*A*	*D*—H···*A*
O1—H1A···N1	0.82	1.80	2.5344 (17)	148
O2—H2A···N2	0.82	1.81	2.5431 (18)	148
C17—H17···O2^i^	0.93	2.56	3.481 (2)	168
C18—H18···O1^ii^	0.93	2.47	3.395 (3)	174
C21—H21···O2^iii^	0.93	2.56	3.492 (2)	175

Symmetry codes: (i) −*x*+1, *y*+1/2, −*z*+1/2; (ii) −*x*+1, −*y*+1, −*z*+1; (iii) −*x*+1, *y*−1/2, −*z*+1/2.
